# Pre-Travel Consultation Without Injury Prevention Is Incomplete

**DOI:** 10.1111/jtm.12041

**Published:** 2013

**Authors:** Shirin Wadhwaniya, Adnan A. Hyder

**Affiliations:** Johns Hopkins International Injury Research Unit, Department of International Health, Johns Hopkins Bloomberg School of Public Health, Baltimore, MD, USA

International travel is fast growing. In 2011, 982 million international tourists traveled around the world to visit friends and relatives, for business, leisure, or other purposes.^1^ While Europe (51%) continues to be a popular tourist destination attracting about half a billion people, Asia and the Pacific (22%) are also gaining popularity.^1^ In 2011, 217 million people traveled to Asia-Pacific and 50 million people traveled to the African region and these are projected to become leading travel destinations in the near future.^1^ This means that more than ever before, more people will be traveling to low and middle income countries (LMICs) of the world.

Over the years, as travel patterns and destinations are changing, travel medicine is attempting to keep pace to reduce risk of diseases and adverse health events and to make travel a healthy and enjoyable experience. With increasing availability of immunizations and prophylactic treatments, a change in morbidity and mortality patterns has been observed among global travelers. Infectious diseases now account for a very small proportion of reported deaths (<2%) among travelers.^2^ Travelers however are now 10 times more likely to die from injuries than from infectious diseases, which presents a relatively new challenge for travel medicine.^2^

## Travel and Injury

Several studies have examined the causes of mortality among travelers and in these studies injuries were found to be a leading cause of preventable deaths; and the most common cause of injury deaths was road traffic injuries (RTIs).^3-7^ RTI was also the major reason to transfer US citizens out of a country after non-fatal injuries.^2^ Other causes of injury deaths among travelers include homicide, drowning, and suicide.^2,4-7^

In 2010, RTIs ranked as the 8th leading cause of death in the world, and in the last decade moved up from the 14th to the 8th leading cause of global years of life lost (YLL).^8^ LMICs account for 90% of the world’s fatal RTIs despite having only half the share (48%) of the world’s vehicles.^9^ Thus, with increasing travel to LMICs, high-income travelers are exposed to a much higher risk of RTI than in their home country (Table 1). For instance, in high-income countries in Europe the fatal RTI rate (12 per 100,000 population) is much lower than in LMICs in the African Region (28.3 per 100,000).^10^

Regional differences in the distribution of fatal injuries among travelers have already been reported. Groenheide and colleagues found that compared to traveling within Europe, the mortality risk from fatal injuries increased by 40 times when traveling to the African or Eastern Mediterranean Region,^3^ while Tonellato and colleagues reported a higher proportion of RTI deaths among travelers visiting LMICs of the Americas.^11^ These findings are reflective of the high burden of fatal RTIs in the LMICs of the world (Table 1).

A larger proportion of RTI deaths are also found to occur outside hospitals indicating severe injuries or limited access to health facilities and emergency medical services.^5^ For example, Tonellato and colleagues found a higher proportion of injury deaths among US citizens abroad compared to injury deaths among US citizens within the country; they also found that US citizens abroad had a higher mortality rate from RTIs compared to local residents.^11^ Similar findings are also reported from sites most frequented by tourists where RTI rates were higher in travelers compared to local residents.^12^ Thus, travelers do not share the same risk of RTIs either with local residents or citizens of their country of origin but in fact have a higher risk of RTIs.

Characterizing those travelers at risk of RTIs is challenging because of lack of data. However, gender is an issue and males are more affected.^4^ This observation is also consistent with data on global and regional patterns of RTIs as well.^10^ These trends are not found only in tourists but international business travelers have also reported increased risk of RTIs abroad. A survey conducted among employees of the World Bank Group reported an incidence of 1 near road-traffic crash per 15 travel missions and 1 road-crash per 175 travel missions.^13^ These rates reflected a much higher risk of RTIs for World Bank employees compared to other diseases.

Of course, behind these numbers are real stories of aspiring young individuals like Aron Sobel, a US medical student who lost his life, along with 22 other passengers while traveling on a bus in Turkey.^14^ His story became the inspiration for establishing the Association for Safe International Road Travel (www.asirt.org). The human toll of such events during travel is immeasurable—lives lost, families affected, and societies deprived of professionals. With more and more young individuals exploring the world through traveling, studying, volunteering, or researching outside their home countries, it is imperative that they are protected from all travel-related harms including injuries.

## Pre-Travel Consultation and Injury

One important strategy for protection is pre-travel consultation, which can play an important role in injury prevention. A pre-travel consultation is expected to include an assessment to identify potential risks at the travel site and from travel itself; risk communication aimed at discussing the risks identified during assessment; and risk management through immunizations, prophylactic medications, and health education.^2^ Health education is an essential but often neglected component of pre-travel consultation; providers tend to focus more on prevention of infectious diseases through vaccination and administration of prophylactic medications.^15-18^ In addition, pre-travel consultations are brief and do not offer enough time to conduct education and health promotion.^16,17^ With international travel soon reaching the 1 billion people traveling per year mark and growing, more effort is needed to explore ways in which injury prevention can be adequately included in pre-travel consultation.

An important prerequisite for communication is risk perception, and if providers and travelers do not perceive injuries as risks during travel they are less likely to discuss these or suggest preventive measures. In this issue of the *Journal of Travel Medicine*, Piotte and colleagues present findings from their study evaluating pre-travel consultation provided by primary care physicians (PCPs) in France.^18^ They present the case of a 25-year-old man traveling alone for a 1-month trek in Peru for whom only 30% of PCPs recommended “repatriation insurance.”^18^ Higher risk of injuries is observed in young men and despite the travel itinerary and age-associated risk, fewer PCPs perceived injuries as a risk. In fact, PCPs were more likely to recommend water, hand hygiene, and use of condoms than injury prevention advice. Travelers themselves may also underestimate the risk of injuries, though this perception may change substantially post-travel.^19^

The higher risk of RTIs among travelers is caused by many reasons: varied mix of traffic, poor road conditions, unfamiliarity with traffic rules, unavailability of road safety measures—helmets, seatbelts, child restraints—adventure-seeking attitude during travel, drinking and driving, speeding, lack of concentration because of exhaustion, jetlag, and cell phone usage when drivings, amongst others.^13^ Some of these factors are preventable and pre-travel consultations can include a focused discussion on road safety measures and provision of resources to seek more specific advice. Clear messages on the risks and how they can be reduced ought to be an important part of pre-travel consults (Table 2).

It has been observed that travelers do not adhere to all the pre-travel advice that they receive for prevention of infectious diseases.^20^ This may turn out to be the case even for injury prevention advice; therefore alternative approaches to communication and development of factual materials will need to be explored. Further research can also be conducted in the future to study if pre-travel injury prevention advice has an effect on injury outcomes among travelers; this will provide a measure of real effectiveness. In the meantime, injuries are a grave risk for travelers and we propose that pre-travel consultations remain incomplete until they include injury prevention.

## Figures and Tables

**Figure:**
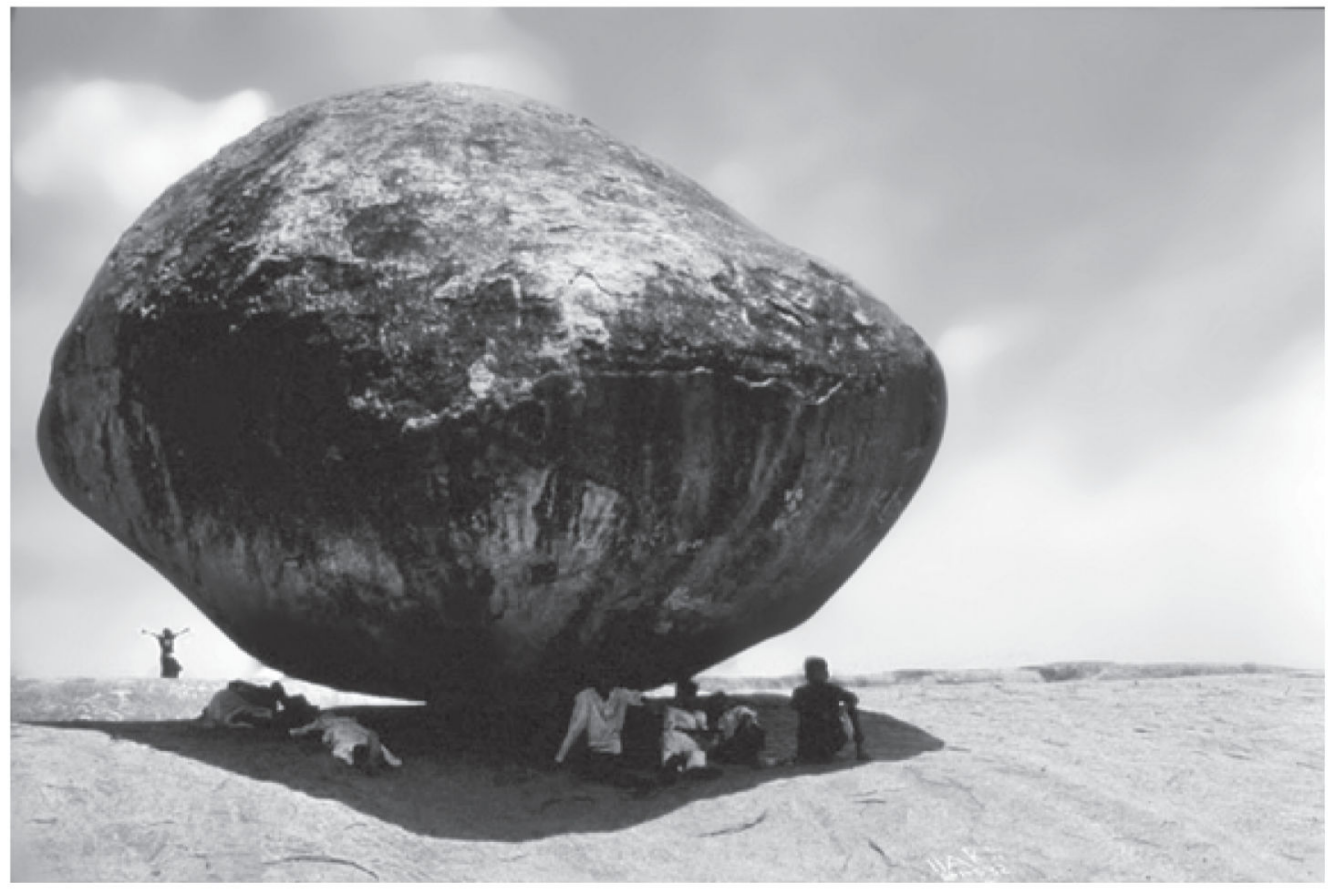
This image has been taken in Mahabalipuram or Mamallapuram (Tamil Nadu, India). It shows a big rounded stone named “Krishna’s butter ball” standing on a slope in apparent poor equilibrium, but in fact has managed to maintain its position for centuries. It can illustrate the importance of the “risk perception” for travelers and persons performing “pre-travel consultations”, an issue that is approached differently in four papers published in this issue, the Editorial by Wadhwaniya and Hyder (pages 217–220), the Original Article by Piotte et al. (pages 221–227), the Letter to the Editor by R. Zimmer and the Response to this letter by R. Zimmermann (pages 269–272). *Photo Credit: Eric Caumes*

**Table 1:** Countries with highest burden of road traffic injuries

	Countries with highest number of road traffic deaths		Countries with highest road traffic death rates	
*Ranking*	Country	Estimated number of road traffic deaths	Country	Estimated road traffic death rate (per 100,000 population)
1	China	220,783	Eritrea	48.4
2	India	196,445	Cook Islands	45.0
3	Nigeria	47,865	Egypt	41.6
4	United States	42,642	Libya	40.5
5	Pakistan	41,494	Afghanistan	39.0
6	Indonesia	37,438	Iraq	38.1
7	Russian Federation	35,972	Angola	37.7
8	Brazil	35,155	Niger	37.7
9	Egypt	31,439	United Arab Emirates	37.1
10	Ethiopia	29,114	Gambia	36.6
11	Iran	25,491	Iran	35.8
12	Mexico	22,103	Mauritania	35.5
13	Democratic Republic of Congo	20,183	Ethiopia	35.0
14	Bangladesh	20,038	Mozambique	34.7
15	Philippines	17,557	Sudan	34.7
16	Thailand	16,240	Tunisia	34.5
17	South Africa	16,113	Guinea-Bissau	34.4
18	Vietnam	14,104	Kenya	34.4
19	Tanzania	13,886	Chad	34.3
20	Sudan	13,362	Tanzania	34.3

*Source*: Global status report on road safety: time for action. Geneva, World Health Organization, 2009. Global Health Observatory Data Repository, World Health Organization, http://apps.who.int/gho/data/?vid=51310

**Table 2:** Examples of pre-travel consultation advice and resources for road travel

As driver:
Get familiarized with local traffic and signs before driving Avoid driving after dark Avoid driving or riding motorized two-wheelers Always use helmet when riding a bicycle or motorcycle Do not drink and drive Follow traffic rules Always use seatbelts and child restraints Do not drive when jetlagged or exhausted
As passenger:
Use registered vehicles only Choose safe modes of transportation Communicate to the driver ifyou are uncomfortable with the driving situation or road conditions
As pedestrian:
Look on both sides before crossing the road Follow traffic rules Use pavement/sidewalk
Other safety measures:
Keep charged cell-phone Keep local emergency phone numbers handy
Resources for more information:
Association for Safe International Road Travel http://www.asirt.org Centers for Disease Control and Prevention http://www.cdc.gov World Health Organization http://www.who.int/en/ US Department ofState http://travel.state.gov
